# Network Archaeology: Uncovering Ancient Networks from Present-Day Interactions

**DOI:** 10.1371/journal.pcbi.1001119

**Published:** 2011-04-14

**Authors:** Saket Navlakha, Carl Kingsford

**Affiliations:** Department of Computer Science and Center for Bioinformatics and Computational Biology, University of Maryland, College Park, Maryland, United States of America; Johns Hopkins University, United States of America

## Abstract

What proteins interacted in a long-extinct ancestor of yeast? How have different members of a protein complex assembled together over time? Our ability to answer such questions has been limited by the unavailability of ancestral protein-protein interaction (PPI) networks. To overcome this limitation, we propose several novel algorithms to reconstruct the growth history of a present-day network. Our likelihood-based method finds a probable previous state of the graph by applying an assumed growth model backwards in time. This approach retains node identities so that the history of individual nodes can be tracked. Using this methodology, we estimate protein ages in the yeast PPI network that are in good agreement with sequence-based estimates of age and with structural features of protein complexes. Further, by comparing the quality of the inferred histories for several different growth models (duplication-mutation with complementarity, forest fire, and preferential attachment), we provide additional evidence that a duplication-based model captures many features of PPI network growth better than models designed to mimic social network growth. From the reconstructed history, we model the arrival time of extant and ancestral interactions and predict that complexes have significantly re-wired over time and that new edges tend to form within existing complexes. We also hypothesize a distribution of per-protein duplication rates, track the change of the network's clustering coefficient, and predict paralogous relationships between extant proteins that are likely to be complementary to the relationships inferred using sequence alone. Finally, we infer plausible parameters for the model, thereby predicting the relative probability of various evolutionary events. The success of these algorithms indicates that parts of the history of the yeast PPI are encoded in its present-day form.

## Author Summary

Many questions about present-day interaction networks could be answered by tracking how the network changed over time. We present a suite of algorithms to uncover an approximate node-by-node and edge-by-edge history of changes of a network when given only a present-day network and a plausible growth model by which it evolved. Our approach tracks the extant network backwards in time by finding high-likelihood previous configurations. Using topology alone, we show we can estimate protein ages and can identify anchor nodes from which proteins have duplicated. Our reconstructed histories also allow us to study how topological properties of the network have changed over time and how interactions and modules may have evolved. Further, we provide another line of evidence indicating that major features of the evolution of the yeast PPI are best captured by a duplication-based model. The study of inferred ancient networks is a novel application of dynamic network analysis that can unveil the evolutionary principles that drive cellular mechanisms. The algorithms presented here will likely also be useful for investigating other ancient, unavailable networks.

## Introduction

Many biological, social, and technological networks are the product of an evolutionary process that has guided their growth. Tracking how networks have changed over time can help us answer questions about why currently observed network structures exist and how they may change in the future [1]. Analyses of network growth dynamics have studied how properties such as node centrality and community structure change over time [1]–[4], how structural patterns have been gained and lost [5], and how information propagates in a network [6].

However, in many cases only a static snapshot of a network is available without a node-by-node or edge-by-edge history of changes. Biology is an archetypical domain where older networks have been lost, as ancestral species have gone extinct or evolved into present-day organisms. For example, while we do have a few protein-protein interaction (PPI) networks from extant organisms, these networks do not form a linear progression and are instead derived from species at the leaves of a phylogenetic tree. Such networks are separated by millions of years of evolution and are insufficient to track changes at a fine level of detail. For social networks, typically only a single current snapshot is available due to privacy concerns or simply because the network was not closely tracked since its inception. This lack of data makes understanding how the network arose difficult.

Often, although we do not know a network's past, we do know a general principle that governs the network's forward growth. Several network growth models have been widely used to explain the emergent features of observed real-world networks [5], [7]–[12]. These models provide an iterative procedure for growing random graphs that exhibit similar topological features (such as the degree distribution and diameter) as a class of real networks. For example, *preferential attachment* has explained many properties of the growing World Wide Web [7]. The *duplication-mutation with complementarity* model was found by Middendorf et al. [13] to be the generative model that best fit the *D. melanogaster* (fruit fly) protein interaction network. The *forest fire* model was shown [10] to produce networks with properties, such as power-law degree distribution, densification, and shrinking diameter, that are similar to the properties of real-world online social networks.

Although these random graph models by themselves have been useful for understanding global changes in the network, a randomly grown network will generally not isomorphically match a target network. This means that the history of a random graph will not correspond to the history of a real network. Hence, forward growth of random networks can only explore properties generic to the model and cannot track an individual, observed node's journey through time. This problem can be avoided, however, if instead of growing a random graph forward according to an evolutionary model, we decompose the actual observed network *backwards* in time, as dictated by the model. The resulting sequence of networks constitute a model-inferred history of the present-day network.

Reconstructing ancestral networks has many applications. The inferred histories can be used to estimate the age of nodes, to model the evolution of interactions (both extant and ancestral), and to track the emergence of prevalent network clusters and motifs [14]. In addition, proposed growth models can be validated by selecting the corresponding history that best matches the known history or other external information. Leskovec et al. [12] explore this idea by computing the likelihood of a model based on how well the model explains each observed edge in a given complete history of the network. This augments judging a model on its ability to reproduce certain global network properties, which by itself can be misleading. As an example, Middendorf et al. [13] found that networks grown forward according to the small-world model [15] reproduced the small-world property characteristic of the *D. melanogaster* PPI network, but did not match the empirical PPI network in other aspects. Leskovec et al. [10] made a similar observation for social network models. Ancestor reconstruction also can be used to down-sample a network to create a realistic but smaller network that preserves key topological properties and node labels. This can be used for faster execution of expensive graph algorithms or for visualization purposes. In the biological network setting, network histories can provide a view of evolution that is complementary to that derived from sequence data alone. In the social network setting, if a network's owner decides to disclose only a single network, successful network reconstruction would allow us to estimate when a particular node entered the network and reproduce its activity since being a member. This could have privacy implications that might warrant the need for additional anonymization or randomization of the network.

Some attempts have been made to find small “seed graphs” from which particular models may have started. Leskovec et al. [11], under the Kronecker model [16], and Hormozdiari et al. [17], under a duplication-based model, found seed graphs that are likely to produce graphs with specified properties. These seed graphs can be thought of as the ancestral graphs at very large timescales, but the techniques to infer them do not generalize to shorter timescales nor do they incorporate node labels. Previous studies of time-varying networks solve related network inference problems, but assume different available data. For example, the use of exponential random graph models [18], [19] and other approaches [20] for inferring dynamic networks requires observed node attributes (e.g. gene expression) at each time point. They are also limited because they use models without a plausible biological mechanism and require the set of nodes to be known at each time point. Wiuf et al. [21] use importance sampling to compute the most likely parameters that gave rise to a PPI network for *C. elegans* according to a duplication-attachment model, but they do not explicitly reconstruct ancient networks. Mithani et al. [22] only model the loss and gain of edges amongst a fixed set of nodes in metabolic networks. There has also been some work on inferring ancestral biological networks using gene trees [23]–[26]. These approaches “play the tape” of duplication instructions encoded in the gene tree backwards. The gene tree provides a sequence-level view of evolutionary history, which should correlate with the network history, but their relationship can also be complementary [27]. Further, gene tree approaches can only capture node arrival and loss (taken directly from the gene tree), do not account for models of edge evolution, and are constrained to only consider trees built per gene family. Network alignment between two extant species has also been used to find conserved network structures, which putatively correspond to ancestral subnetworks [28]–[30]. However, these methods do not model the evolution of interactions, or do so using heuristic measures. Finally, the study of ancestral biological sequences has a long history, supported by extensive work in phylogenetics [31]. Sequence reconstructions have been used to associate genes with their function, understand how the environment has affected genomes, and to determine the amino acid composition of ancestral life. Answering similar questions in the network setting, however, requires significantly different methodologies.

Here, we propose a likelihood-based framework for reconstructing predecessor graphs at many timescales for the preferential attachment (PA), duplication-mutation with complementarity (DMC), and forest fire (FF) network growth models. Our efficient greedy heuristic finds high likelihood ancestral graphs using only topological information and preserves the identity of each node, allowing the history of each node and edge to be tracked. To gain confidence in the procedure, we show using simulated data that network histories can be inferred for these models even in the presence of some network noise.

When applied to a protein-protein interaction (PPI) network for *Saccharomyces cerevisiae*, the inferred, DMC-based history agrees with many previously predicted features of PPI network evolution. It accurately estimates the sequence-derived age of a protein when using the DMC model, and it identifies known functionally related proteins to be the product of duplication events. In addition, it predicts older proteins to be more likely to be at the core of protein complexes, confirming a result obtained via other means [32].

By comparing the predicted protein ages using different models, we further confirm DMC as a better mechanism to model the growth of PPI networks [13] compared to the PA model [7] or the FF model [10], which are designed for web and social networks. Conversely, when applied to a social network (derived from the music service Last.fm), the DMC model does not produce as accurate an ancestral network reconstruction as that of PA. The FF model also outperforms DMC in the social network context at the task of identifying users who putatively mediated the network's growth by attracting new members to join the service. Thus, models of social network evolution do not transfer well to biological networks, and vice versa — a well-studied and expected notion that we confirm through alternative means.

We also used our reconstructed history of the PPI network to make several novel predictions. For example, we estimate the arrival time of extant and ancestral interactions and predict that newly added extant edges often connect proteins within the same complex and that modules have recently gained many peripheral units. The history can also be used to track the change of network topological properties over time, such as the clustering coefficient, which we find has been decreasing in recent evolution. Analysis of the duplication rates over the inferred history suggests that proteins with fewer extant interactions have been involved in the largest number of duplication events, which is in broad agreement with existing belief that proteins with many interactions evolve more slowly [33], [34]. In addition, the reconstruction predicts paralogous relationships between proteins that are strongly implied by network topology and which partially agree with sequence-based estimates. Thus, the reconstructed history makes a number of detailed predictions about the relative order of events in the evolution of the yeast PPI, many of which correlate with known biology and many of which are novel.

The ability of these algorithms to reconstruct significant features of a network's history from topology alone further confirms the utility of models of network evolution, suggests an alternative approach to validate growth models, and ultimately reveals that some of the history of a network is encoded in a single snapshot.

## Results

### Network reconstruction algorithms

Suppose an observable, present-day network is the product of a growth process that involved a series of operations specified by a model 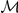 (such as preferential attachment). The model 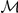 gives us a way to grow the network forward. We see now how this process can be reversed to find a precursor network.

We start with a snapshot of the network 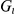 at time 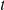, and would like to infer what the network looked like at time 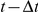. One approach to find the precursor network 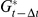 is to find the maximum *a posteriori* choice:
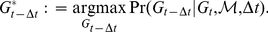
(1)


In other words, we seek the most probable ancestral graph 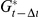, given that the observed graph 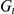 has been generated from it in time 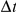 under the assumed model 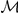. Our goal is to find an entire most probable sequence of graphs 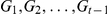 that led to the given network 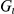 under model 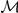.

Because the space of possible ancestral graphs grows exponentially with 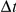 for all reasonable models, Equation (1) poses a challenging computational problem. A heuristic simplification that makes inference somewhat more feasible is to set 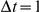 and greedily reverse only a single step of the evolutionary model. While this will no longer find the maximum a posteriori estimate for larger 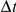, it is much more tractable. Repeated application of the single-step reversal process can derive older networks. We make the first-order Markov model assumption (also made by the growth models) that 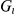 only depends on 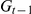. In this case, applying Bayes' theorem, we can rewrite Equation (1) as:
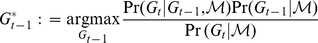
(2)

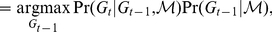
(3)where the last equality follows because the denominator is constant over the range of the 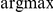. This formulation has the advantage that the model 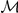 is being run forward as intended. The formulation also has the advantage that the prior 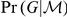 in Equation (3) can be used to guide the choice of 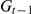. Computing 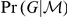 exactly for various models is an interesting computational problem in its own right [35] with a number of applications beyond ancestral network reconstruction. For computational simplicity, here we assume a uniform prior and therefore consider the term a constant.

The ancestral reconstruction algorithm chooses the predecessor graph with the largest conditional probability 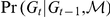 by searching over all possible predecessors graphs, 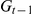. In all models we consider, a single new node enters the network in each time step and connects to some existing nodes in the network. In the DMC and FF models, the new node performs a link-copying procedure from a randomly chosen *anchor node*. Finding the most probable predecessor graph therefore corresponds to finding and removing the most recently added node, identifying the node it duplicated from (if applicable to the model), and adding or removing edges that were modified when the most recently added node entered the network. In the next sections, we explain how to do these steps efficiently for the DMC, FF, and PA growth models.

### The duplication-mutation with complementarity (DMC) model

The DMC model is based on the duplication-divergence principle in which gene duplication produces a functionally equivalent protein, which is followed by divergence when the pair specialize into subtasks. Middendorf et al. [13] and Vazquez et al. [8] have provided support and an evolutionary basis for the general duplication model, which has been widely studied as a route by which organism complexity has increased [9], [36]–[38]. Though some questions remain about its exact role in evolution [32], the DMC model appears to have a computational and biological basis for reproducing many features of real protein interaction networks.

The forward DMC model begins with a simple, connected two-node graph. In each step, growth proceeds as follows:

1. Node 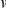 enters the network by duplicating from a random anchor node 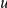. Initially, 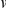 is connected to all of 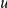 's neighbors (and to no other nodes).
2. For each neighbor 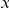 of 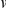, decide to modify the edge or its compliment with probability 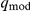. If the edge is to be modified, delete either edge 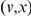 or 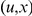 by the flip of a fair coin.
3. Add edge 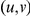 with probability 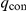.

**Figure 1:**
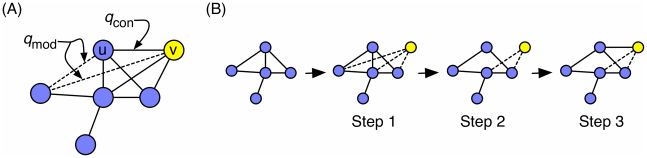
Duplication-mutation with complementarity (DMC). (A) The probabilities governing the DMC model. (B) An example iteration of the DMC model.

A schematic of the growth process is shown in Figure 1.

To reverse DMC, given the two model parameters 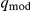 and 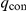, we attempt to find the node that most recently entered the current network 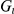, along with the node in 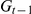 from which it duplicated (its anchor). Merging this pair produces the most likely predecessor graph of Equation (3). Formally, 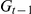 is formed by merging:
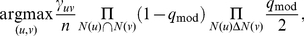
(4)where 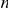 is the number of nodes in 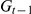, 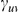 equals 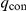 if 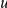 and 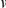 are connected by an edge and 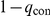 if not, 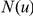 denotes neighbors of node 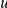, and the pairs 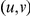 range over all pairs of nodes in 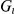. The expression inside the 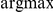 of Equation (4) corresponds to 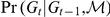, which is what we are trying to maximize in Equation (3) by selecting 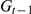. The 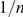 factor gives the probability that node 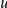 was chosen as the node to be duplicated. The first product considers the common neighbors between the two nodes. In the DMC model, a node and its duplicate ultimately share a neighbor 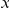 if 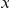 was not modified in step 2 of the model. The probability of such an event is 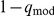. The second product involves the nodes that are neighbors of 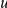 or 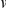 but not both (symmetric difference of 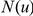 and 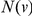). Each such neighbor exists with probability 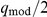.

If 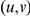 is a pair that maximizes Equation (4), the predecessor graph 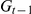 is formed by removing either 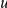 or 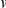. Let 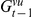 correspond to the graph where 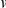 is removed. Due to symmetry, both 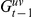 and 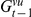 yield the same likelihood in Equation (4), and thus we are forced to arbitrarily decide which node to remove. Assume we randomly choose to remove 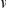; then 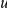 gains edges to all nodes in 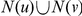 that it does not already have an edge to. This is because, according to the forward growth model, 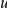 originally had these edges prior to the duplication event of 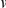 and subsequent divergence.

Any pair of nodes in 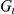 could correspond to the most recently duplicated pair, including pairs with no common neighbors (which would happen if after duplication all edges were modified in step 2 of the model). Thus, all 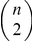 pairs of nodes must be considered in Equation (4).

### The forest fire (FF) model

The forest fire (FF) model was suggested by Leskovec et al. [10] to grow networks that mimic certain properties of social networks. These properties include power-law degree, eigenvalue, and eigenvector distributions, community structure, a shrinking diameter, and network densification.

The forward FF model begins with a simple, connected two-node graph. In the undirected case, in each step, growth proceeds according to the following procedure with parameter 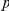:

1. Node 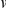 enters the network, selects a random anchor node 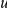, and links to it.
2. Node 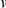 randomly chooses 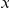 neighbors of 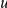 and links to them, where 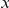 is an integer chosen from a geometric distribution with mean 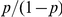. These vertices are flagged as active vertices.
3. Set 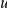 to each active vertex and recursively apply step 2. Node 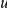 becomes non-active. Stop when no active vertices remain.

To prevent cycling, a node cannot be visited more than once. The process can be thought of as a fire that starts at node 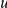 and probabilistically moves forward to some nodes in 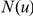, then some nodes in 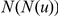, etc. until the spreading ceases. This version of the model only contains one parameter: 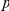, the burning probability. As in the DMC model, the reversal process for the FF model attempts to find the node in the current network 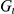 that most recently entered the network, along with its anchor.

Unfortunately, it appears to be difficult to write down an analytic expression computing the likelihood of 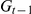. The main challenge is that for every 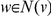 we need to find the likely paths through which the fire spread from 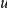 to 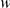. However, these paths are not independent, and therefore cannot be considered separately. Analytic evaluation of the global network properties produced by the model also appears to be difficult [10]. Instead, we compute the likelihood of 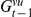 via simulation as follows:

**Forest Fire Simulation Procedure.** We assume 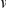 does not exist in the network and simulate the FF model starting from a candidate anchor 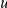. Each simulation produces a set of visited nodes 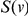 corresponding to candidate neighbors of 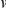. We use the fraction of simulations in which 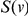 exactly equals 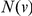 as a proxy for the likelihood of 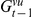.

In the FF model, the likelihood of 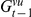 does not necessarily equal that of 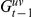 because a forest fire starting at 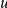 could visit different nodes than a forest fire starting at 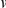. The advantage of non-symmetry here is that there is no uncertainty regarding which node to remove. Also, unlike the DMC model, all candidate node/anchor pairs must have an edge between them (because of step 1 of the model). After identifying the node/anchor pair 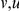 that yields the most likely 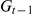, we remove 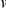 and all its edges from the graph. No edges need to be added to 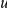 as per the model.

Leskovec et al. [10] also propose a directed version of the FF model where the fire can also spread to incoming edges with a lower probability. Interestingly, reversing the directed FF model is much easier than the undirected case because the node that most recently entered the network must have exactly 0 incoming edges. Choosing which of the nodes with a 0 in-degree to remove first can be difficult because several nodes could have been added to distant, independent locations in the graph in separate steps. A node's anchor, however, can still be determined using our approach.

### The preferential attachment (PA) model

The preferential attachment (PA) model was originally investigated by Simon [39] and de Solla Price [40] and was later proposed by Barabási et al. [7] as a mechanism to emulate the growth of the Web. It follows the premise that new pages make popular pages more popular over time by linking to them preferentially. We consider the linear version of the PA model, which has been shown to correspond closely with the growth of citation networks and online social networks [12], [41].

The PA model begins with a clique of 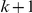 nodes. In each step 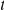, forward growth proceeds with parameter 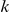 as follows:

1. Create a probability distribution histogram, where each node 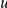 is assigned probability 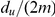, where 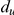 is the degree of 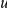 and 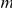 is the total number of edges in 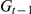.
2. Choose 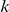 nodes according to the distribution.
3. Node 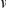 enters the network and links to the 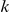 nodes from step 2.

Unlike the DMC and FF models, there is no notion of a node anchor in PA. A new node simply enters the network in each step and preferentially attaches to nodes with high degree. The most recently added node must be of minimum degree in 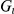 because all nodes start with degree 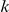 and can only gain edges over time. Let 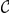 be the set of nodes with minimum degree. To produce 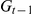, we choose a node to remove from among the nodes in 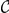 by computing:
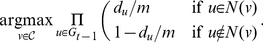
(5)


The two cases in the product correspond to whether edge 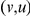 exists. The degree of 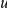 in 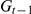 can vary depending on which candidate node 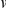 is being considered for removal from 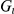. Taking logs and simplifying turns (5) into:
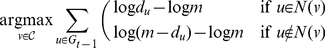
(6)

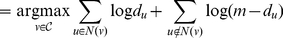
(7)


The 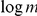 terms in Equation (6) can be ignored because they sum to 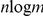 which is a constant over all candidate nodes. Equation (7) seeks to remove the node with minimal degree that links to the nodes of highest degree. If all nodes with minimal degree have an undefined likelihood, we remove a random node from the entire graph. The likelihood is independent of 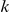.

### The reconstruction algorithms

**Figure 2:**
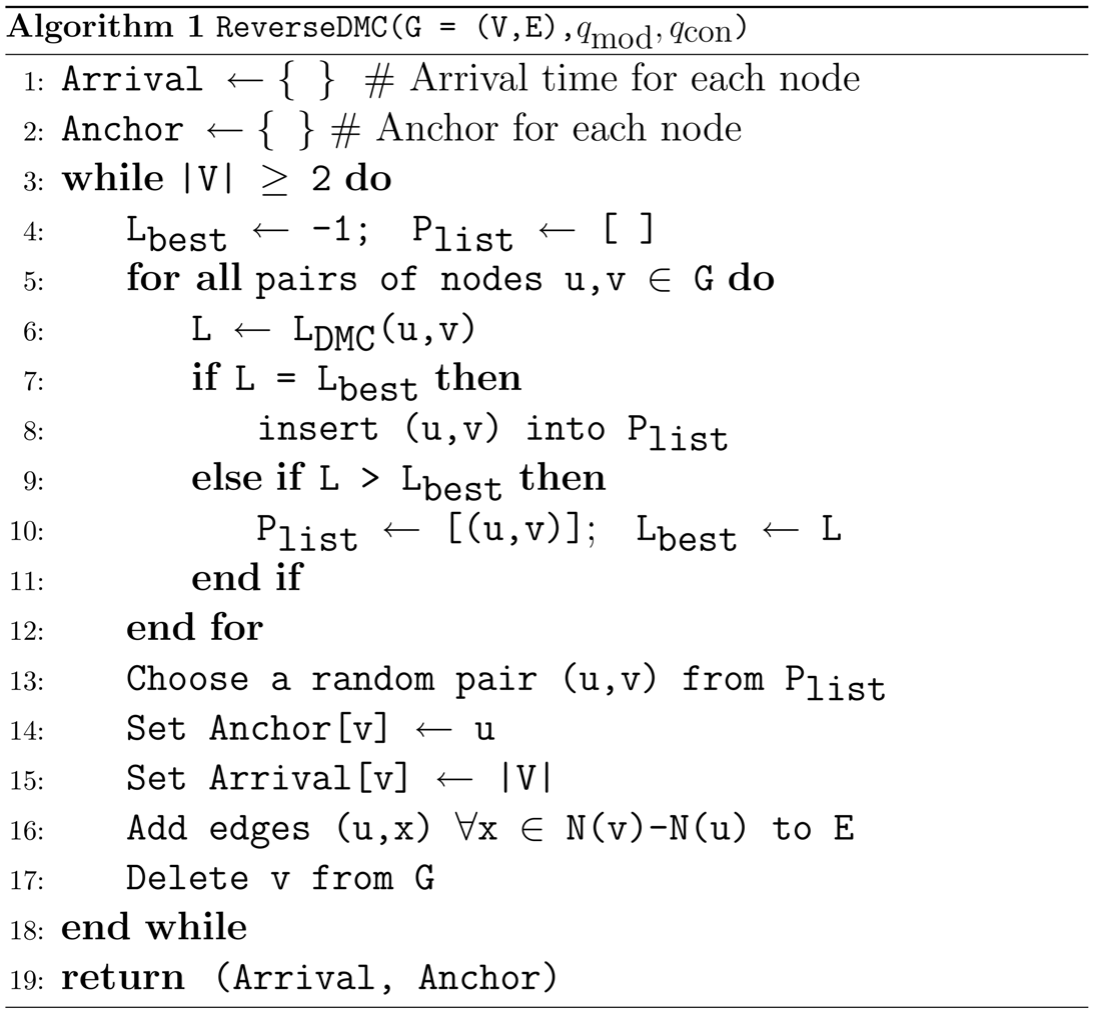
Pseudocode for reversing a network using the DMC model.

The expression inside of the 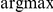 of Equation (4) for DMC defines a score for pairs of nodes. The corresponding score for PA is given in Equation (7) and for FF in the simulation procedure. These scores corresponds to the conditional probability 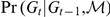 for each model. Let 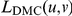, 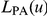, and 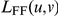 denote these computed scores. To reverse each model, we iteratively search for the nodes that maximize these scores. If there are ties, we randomly choose among them. We continue this process until only a single node remains in the graph. For example, Algorithm 1 (Figure 2) gives the pseudocode for reversing a network using the DMC model. The algorithm takes a static, present-day graph 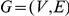 and values for parameters 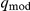 and 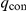.

The likelihood for each pair of nodes can be stored in a matrix, leading to an overall space complexity of 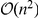. In the case of a clique graph, the likelihood of every pair of nodes must be recomputed in each step, leading to a worst case time complexity of 
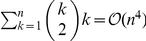
. In general, only the likelihoods of pairs containing the merged node and its neighbors need to be recomputed after each step, which, for real-world (sparse) graphs, leads to a much more efficient algorithm (e.g. for the PPI network, only 0.0003% of the worst-case number of updates were required).

Algorithm 1 (Figure 2) must be changed slightly for the FF and PA models. For the FF model, the differences are: (1) 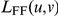 is used instead of 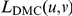; and (2) the for-loop is over all pairs of nodes connected by an edge. For the PA model: (1) 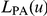 is used; and (2) the for loop is over all nodes instead of all pairs of nodes; and (3) no anchor is stored. For both FF and PA no new edges are added to 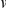 after node 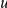 is deleted.

### Model reversibility using the greedy likelihood algorithm

We first tested the algorithms in situations where the evolutionary history is completely known. This allows us to assess the performance of the greedy likelihood algorithm and to compare the reversibility of various network models. For each model (and choice of parameters), we grew 100-node networks forward according to the model, and then supplied only the final network 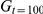 to our algorithm to reconstruct its history. We repeated this process 1000 times and averaged the results for each combination.

**Figure 3:**
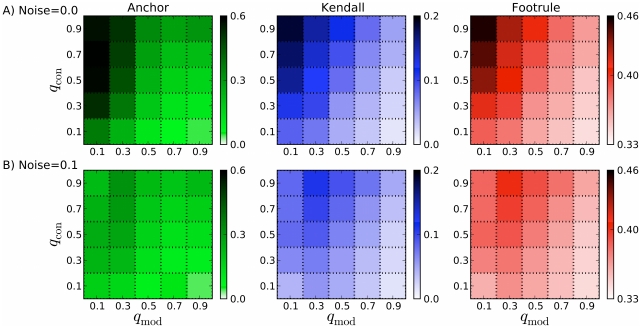
Accuracy of node arrival times and node anchors using the DMC model. The 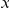 - and 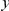 -axes show the DMC parameters (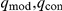) used to grow the synthetic network forward. Each parameter varies from 0.1–0.9 in steps of 0.2. The intensity of each cell in the heatmap represents the quality of the reconstruction validation measure (Anchor, Kendall, Footrule) under optimal reverse parameters. (A) and (B) show results under varying levels of noise. Error bars (not shown) indicate a standard deviation of roughly 7–8% for Kendall and 4–5% for Footrule (over 1000 trials). For many DMC-grown synthetic networks, accurate reconstruction is possible.

For the DMC model under realistic choices of 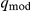 and 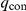, almost 60% (std = 7%) of the node/anchor relationships inferred are correct if the optimal choice of 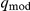 and 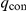 parameters are used in the reconstruction process. Figure 3A plots the performance of three validation measures for 25 combinations of 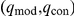 model parameters (see Methods). DMC-grown graphs are generally difficult to reverse because edges can be modified over time; thus, if an incorrect node/anchor pair is merged, new edges will be added to the graph that were never originally present, which can have downstream effects on inference. Still, both the Spearman's footrule and Kendall's 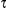 measures of arrival-time correlation indicate that we can order nodes correctly significantly better than random starting from the final graph alone.

Reversibility varies drastically depending on the DMC model parameters used to grow the network forward. Naturally, increasing 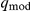 induces more random changes in the network, which makes it more difficult to reverse the evolution. Conversely, as 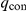 increases, the history generally becomes easier to reverse because more nodes are directly connected to the node from which they duplicated.

**Figure 4:**
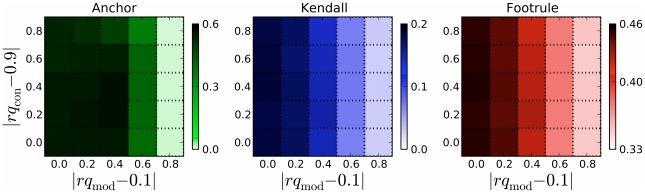
Accuracy of node arrival times and node anchors when reverse parameters are not known. Synthetic DMC-grown networks were constructed using 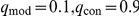 and reversed using all 25 combinations of reversal parameters. The 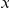 - and 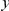 -axes show the difference between the reversal parameters (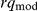 and 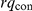, respectively) and the forward parameters (0.1 and 0.9, respectively). The intensity of each cell in the heatmap represents the quality of the reconstruction validation measure with standard deviation lying between 1–7% for Anchor, 7–8% for Kendall, and 4–5% for Footrule. Accurate histories can be inferred as long as reverse parameters (in particular, 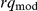) are in the rough range of the forward parameters.

Performance also depends on the match between the values of 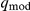 and 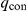 used to grow the network forward and those used to reverse the history (Figure 4). However, even if the forward parameters are not known exactly, it is feasible to reconstruct a meaningful history if the reversal parameters are chosen to be approximately equal to the forward parameters. There is often a hard transition at 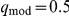 or 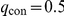 when the bias towards having an edge and not having an edge tips to one side or the other. Though optimal performance can correspond to reversing a network with the same parameters used to grow the network, this need not be the case. For example, suppose 30% of all nodes have edges to their anchors. This does not imply that setting 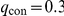 will work best because the true pair sought will likely not be connected and hence even lower values of 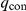 may lead to a more accurate reconstruction.

**Figure 5:**
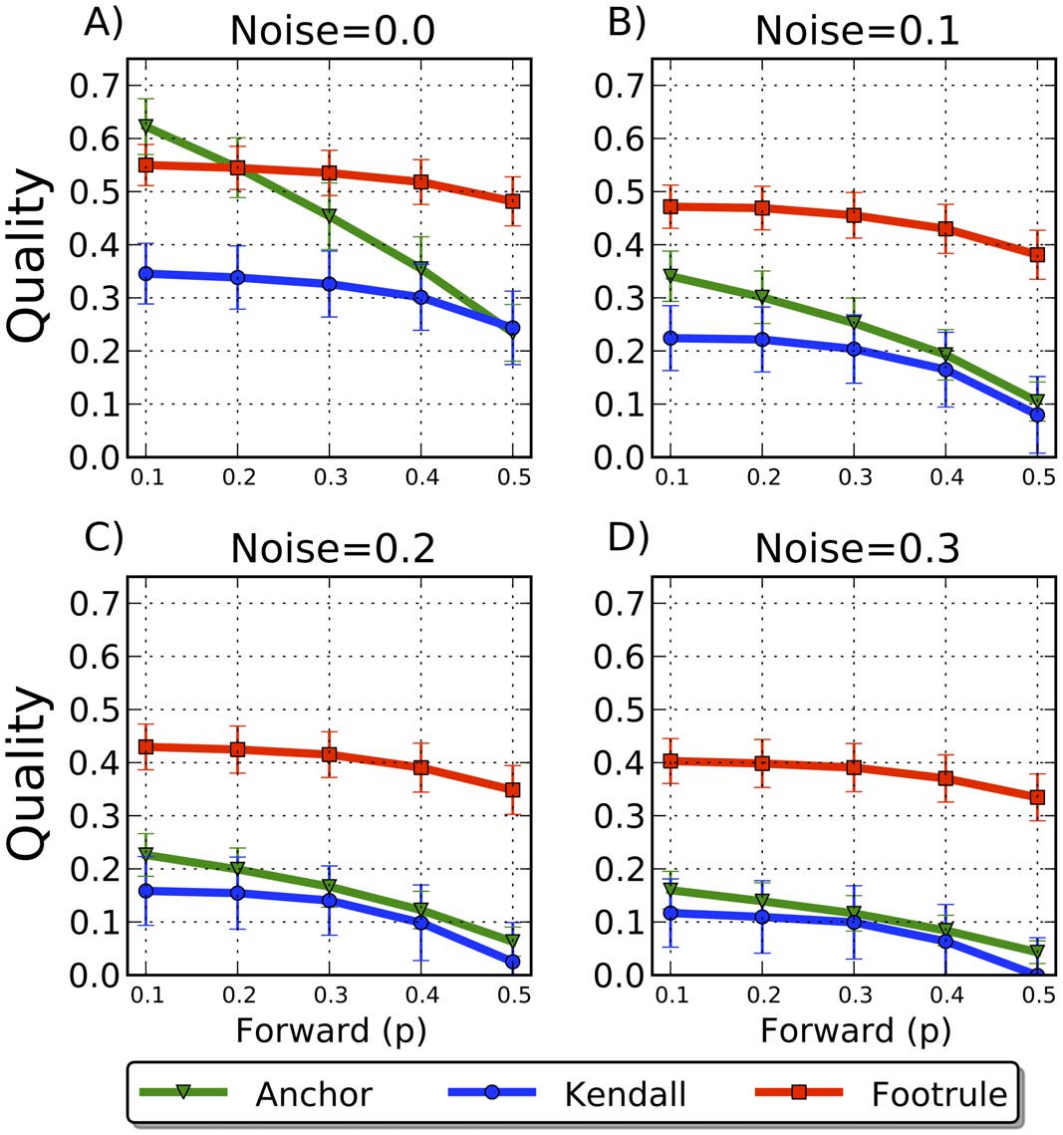
Accuracy of arrival times and node anchors using the forest fire model. (A–D) The 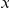 -axis shows the FF parameter (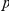) used to grow the synthetic network forward. (Values of parameter 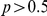 resulted in mostly clique-like networks.) The 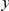 -axis shows the quality of the 3 reconstruction validation measures under optimal reverse parameters (bars show standard deviation over 1000 trials). All FF-based reconstructions are significantly better than random reconstructions, even when 30% of true edges are replaced by random edges.

We performed the same synthetic-data experiments using the forest fire model for varying values of the parameter 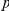, which controls the spread of the fire, ranging from 0.1 to 0.5. Figure 5A shows that between 25% and 64% of anchor relationships can be correctly identified, and that the estimated node arrival ordering resembles the true arrival order. As 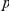 increases, performance of all measures tends to decrease. This is because as 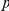 increases, the degree of each node increases, thus making it more difficult to pick out the correct anchor from among the set of neighbors. In general, it is difficult to predict all arrival times correctly because unrelated duplications could occur in successive steps in completely different parts of the graph.

**Figure 6:**
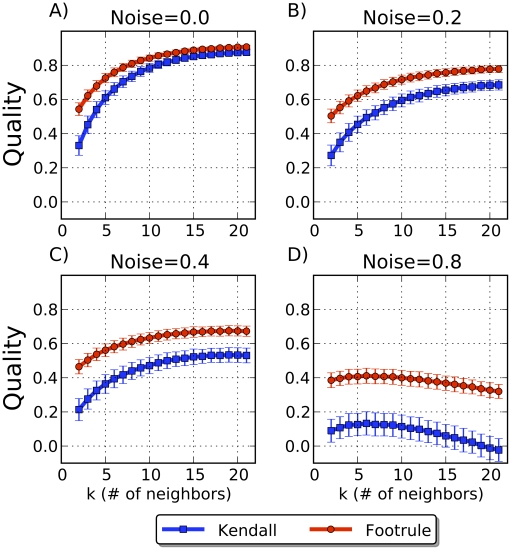
Agreement with arrival times using the preferential attachment model. (A–D) The 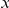 -axis shows the PA parameter (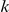) used to grow the synthetic network forward. The 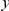 -axis shows the quality of the 3 reconstruction validation measures (bars show standard deviation over 1000 trials). Compared to the DMC and FF models, the PA model is easiest to reverse, even in the presence of substantial noise.

Finally, we grew 100-node networks using the linear preferential attachment model for various choices of parameter 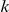, the number of neighbors to which a new node initially connects (Figure 6). Of the three models we consider, PA is the most easily reversible. As 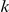 increases, it becomes easier to distinguish amongst low degree nodes connected to hubs because there is less statistical variation in the forward process. This allows more opportunity for older and newer nodes to differentiate themselves from one another, and hence the network becomes easier to reverse. Figure 6A shows that for the PA model we can achieve Kendall 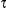 values of over 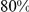 higher than random when 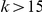. In the PA model, a new node does not choose an anchor node to copy links from so only the arrival-time validation measures are applicable.

### Effect of deviation from the assumed model

To gauge robustness to deviations from the growth model, we repeated the experiments on synthetic data after randomly replacing some percentage of edges in the final graph with new edges. Under all models, reconstruction quality generally suffers from a noisy view of the present-day graph but meaningful histories can still be recovered.

DMC is the most sensitive to the addition of noise (Figure 3B), while PA is by far the most resilient to noise. Even when 80% of the true edges are replaced with random edges, nearly turning the graph into a random graph, reversibility of PA can still be better than random (Figure 6D). DMC can tolerate noise up to 30% before returning essentially random reconstructions. The robustness of the forest fire model lies in between DMC and PA (Figure 5B–D).

Node deletion is a prevalent phenomena in many real-world networks, such as biological networks (which experience gene loss) and online social networks (in which users can delete their accounts). However, deletion is typically not modeled by standard growth mechanisms. To study the effect of deletion on reconstruction, we modified the DMC process so that (in addition to a new node being added) in every step, with probability 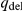, a random existing node is chosen and removed from the graph. Thus the number of nodes in the graph after one iteration can remain constant. This presents an additional challenge to reconstruction because deleted nodes might have been anchors of extant nodes. Upon deletion, this relationship is lost.

We experimented with this modified DMC model using our DMC reconstruction algorithm and found that accuracy degrades smoothly as 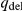 increases (Table 1). At low values of 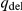, only a few nodes are deleted which implies that the dynamics of the past are still closely reflected in the extant network. For example, at 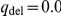, 37.9% of true anchor subtrees are recovered exactly and this only drops to 28.1% at 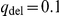. However, at 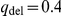, only 13.0% of anchor subtrees are recovered, and the Kendall 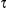 ordering has declined from 11.0% (at no deletion) to 2.6%. The existence of node deletion implies that our reconstructed networks will likely only represent a subsample of true ancestral networks. However, if the relative percentage of deletion is low, significant features of ancient networks can still be recovered.

**Table 1:** The effect of node deletion on reconstruction quality.

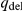	Kendall	Footrule	Anchor
0.0	11.0	40.1	37.9
0.1	9.3	39.2	28.1
0.2	6.7	37.4	22.0
0.3	6.0	37.0	18.4
0.4	2.6	35.1	13.0
0.5	1.4	34.0	10.2

Column headings show the 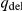 value used in the modified DMC model and the three reconstruction quality measures. Kendall and Footrule are only computed among extant nodes. The other DMC parameters are fixed to 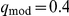 and 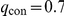. As 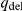 increases, more nodes are lost in the forward growth procedure. This degrades the accuracy of reconstruction because the algorithm is forced to explain interaction partners from anchors that do not exist in 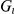. This results in incorrect merges and incorrect ancestral edges. Nonetheless, the algorithm can tolerate deletion at low values of 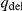. In particular, when 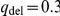 (i.e. in each step, with probability 0.3, a random node is deleted), the Kendall 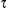 and Anchor quality measures decreases by only half of their original values.

Mis-identifying the model that was used to grow the network can also significantly reduce the quality of the inferred history (Table 2). This degradation in performance can be exploited in conjunction with known node arrival times to select the most plausible model from among a set of network growth models. To verify this, we grew networks forward using each model and reversed it with the other models. In most cases, we found that reversing the network with the model used to grow it forward resulted in optimal performance. For example, for DMC-grown networks 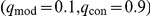, a reversal using DMC results in a 55.6% anchor score vs. 1.8% for FF. The low 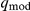 value implies that a node has many reasonable anchors, between which FF cannot easily distinguish. FF and PA also have Footrule scores that are at least 10% less than DMC. PA performed poorly because nodes with late arrival times under DMC can duplicate from hubs and immediately have a high degree. This indicates that reversing DMC-grown networks involves more than removing low-degree nodes. As 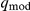 increases, the difference is less pronounced, but the trend still holds.

**Table 2:** Validating network growth models via the confusion matrix.

	DMC(0.1,0.9)	DMC(0.5,0.5)	FF(0.2)	PA(5)	PA(15)
Reverse DMC	**55.6/45.5**	**24.4/38.3**	49.5/41.7	–/58.8	–/64.0
Reverse FF	1.8/33.1	10.7/37.2	**54.5**/54.5	–/28.4	–/24.4
Reverse PA	–/35.0	–/35.0	–/50.6	–/72.6	–**/88.9**
Node degree	–/39.3	–/38.1	–/**59.2**	–**/75.2**	/–85.5
Centrality	–/39.2	–/37.9	–/57.5	–/74.9	–/85.3

Column headings show the model and parameters used to grow the random graph forward. Row labels show the model used in the reversal (assuming optimal parameters). For the node degree reconstruction (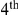 row), we removed nodes in increasing order of their degree in the extant network (nodes with the same degree were ordered randomly). For the centrality reconstruction (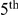 row), we removed nodes in decreasing order of their closeness centrality in the extant network. Each cell contains Anchor/Footrule scores (PA, node degree, and centrality do not generate Anchor scores). Performance was averaged over 1000 runs. Bolded cells indicate best performance. For example, for DMC random graphs with 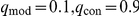, reversing with FF produces a 33.1% Footrule score compared to a 45.5% score when the graph is reversed with DMC itself. The non-model-based heuristics produce good age-estimates when applied to models where degree is known to be correlated with age (FF and PA) as is expected; however, the downside to these approaches is that they do not produce a likelihood estimate for ancestral graphs, nor do they predict node anchors. For identifying anchors and for DMC age estimates, reversing with the model used to grow the graph forward resulted in the best performance.

Similarly, random graphs grown forward using FF (PA) are best reversed using FF (PA) as opposed to the other models. For PA, this is because DMC and FF seek, for each node, a single anchor from which the observed links can be explained. With PA, however, a node can have neighbors that are far apart in the network.

Non-model-based heuristic reconstructions based solely on degree or centrality (Table 2) can perform well when degree strongly implies age (as is the case for FF and PA random graphs). This suggests that additional heuristics might improve our greedy reconstruction algorithms. However, heuristics alone are limiting because they are not driven by a formal mechanism of evolution, they do not predict node anchors, and they do not produce a likelihood estimate for ancestral graphs. Further, even when age is strongly correlated with degree, the likelihood-based procedure can be more accurate. For example, for PA with 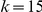, reversing with the PA likelihood algorithm yields a Kendall 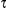 value of 88.9% compared to 85.5% using node degree.

### Recovery of ancient protein interaction networks

**Figure 7:**
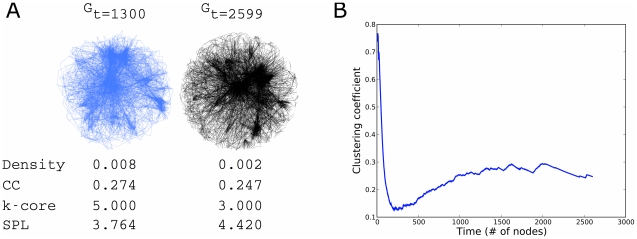
Comparing extant and ancient networks. (A) Visualization [59] of the extant PPI network (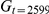) and an ancestral version (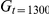). The density, clustering coefficient (CC), average shortest path length (SPL), and average 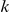 -core number are shown for each network. The ancient network is considerably denser than the extant network. (B) The change in clustering coefficient over time in the yeast network history. Recent evolution (after time step 2000) reveals a trend of decreasing modularity, perhaps due to the addition of peripheral units to existing complexes or pathways. Older evolution (prior to time step 2000, excluding the initial effect of small networks) shows an increasing modularity, suggesting that new clusters were emerging. Other methods have found evidence for both increasing [26] and decreasing [8] clustering coefficient over time.

We obtained a high-confidence protein-protein interaction (PPI) network for the yeast *S. cerevisiae* from the IntAct database [42]. The network contains 2,599 proteins (nodes) and 8,275 physical interactions between them. We applied the reversal algorithm for 2,599 steps to estimate a complete history of the growth of the network. Figure 7A shows the original network (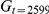) and an inferred ancestral network with 1,300 nodes (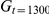).

Because PPI networks from the past are unavailable, we do not directly have true node arrival times to which we can compare. Instead, we estimate protein arrival times using sequence-based homology under the assumption that proteins that have emerged after yeast diverged from other species will have fewer orthologs in these distantly related organisms [43]. In particular, we obtained data for the occurrence of orthologs of yeast proteins in 6 eukaryotes (*A. thaliana*, *C. elegans*, *D. melanogaster*, *H. sapiens*, *S. pombe*, and *E. cuniculi*) from the Clusters of Orthologous Genes database [44]. The number of species for which an ortholog was present was used as a proxy for the arrival time. We grouped proteins into 6 classes and computed a class-based Kendall 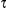 value amongst proteins in different classes. A pair 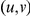 was considered correctly ordered if 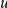 was predicted to arrive before 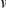 and if 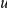 has more orthologs than 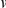; otherwise the pair was considered incorrectly ordered. Although the precise definition of an ortholog is debatable, the COG classes provide a rough benchmark to gauge our temporal orderings.

**Figure 8:**
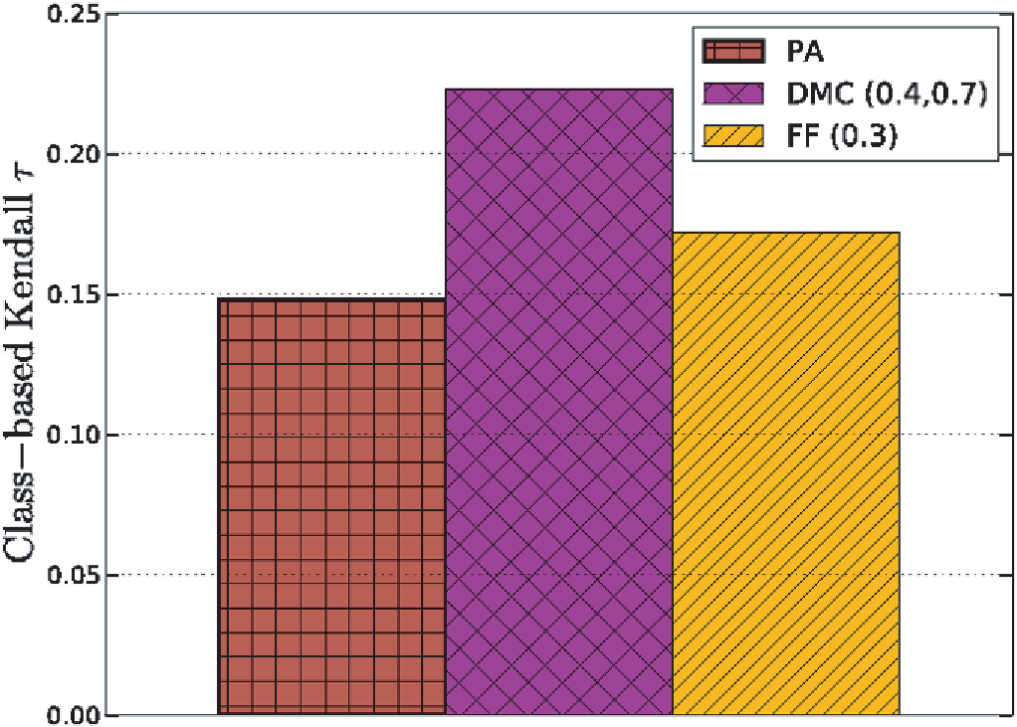
Predicting protein age groups by reversing the DMC and FF models on a real PPI network for *S. cerevisiae*. The 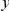 -axis shows the class-based Kendall 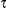 value of the predicted ordering. The DMC model more accurately orders the proteins in the classes compared to FF and PA.

Reversing the network using the DMC model produced an estimated node arrival order in greater concordance with the orthology-based estimates of protein age than either the FF or PA models. Figure 8 shows the class-based Kendall 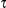 value for proteins in the 6 age classes for all three models. The results shown are the best for each model over the tested parameter space and thus represent the limit of performance using the proposed algorithm. The DMC model more accurately determines the relative ordering of proteins in the age classes than the FF or PA histories (*P* 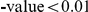 compared to a random reconstruction and after Bonferroni correcting for optimal parameter usage). This provides additional evidence [13] that a duplication-based model is a better fit for PPI networks than models such as FF and PA inspired by social networks.

### Estimation of parameters governing PPI network growth

The parameters that produced the history that best matched the sequence-based estimates of protein ages provide hints about the relative importance of various processes in network growth. For DMC applied to the PPI, the best performance was typically achieved with low-to-medium values of 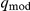 and medium-to-high values of 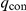. We can use these as estimates of the probability that an interaction is modified following a gene duplication ( 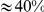) and the probability that two duplicated genes interact (high, as also found elsewhere [45]–[47], though lower values have also been suggested [8]).

Interestingly, the optimal FF and DMC parameters create models that have many similarities. Optimal performance was obtained for the FF model with parameter 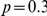, which implies that both the anchor and the arriving node will have similar neighborhoods because the simulated fire likely does not spread far beyond the immediate neighbors of the anchor. The property of similar neighborhoods is also implied by duplication step of DMC coupled with the moderate value of 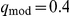. Further, in the FF model the arriving node is always linked to its anchor, and the high value of 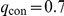 causes this to frequently happen in the DMC model as well. Thus, based on their agreement with sequence-based estimates of protein arrival times, two independent and very different base models both suggest that proteins should very frequently interact with the protein from which they duplicated, and that the new node should primarily interact with neighbors of their anchors.

The actual likelihood values obtained from Equation (4) for DMC also hint at the plausibility of a reconstruction. For the PPI network, the ratio of log-likelihoods between our inferred history and a random reconstruction is 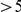, which means that the former is much more likely than the latter. Likelihoods can also be used to select parameter values. For example, the likelihood of the reconstruction with 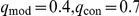 was 2.6 times higher than the (much poorer) reconstruction obtained using 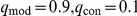. Parameters near the optimal settings also have very similar likelihoods, as expected (e.g. changing from 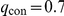 to 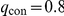 with the same 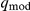 results in a likelihood ratio of 1.01 between the two parameter choices).

Using the optimal 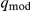 and 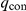 values, we found that in 67% of the inference steps, there is a tie among at least two pairs of nodes with equal likelihood. However, choosing randomly amongst these pairs alters the class-based Kendall 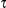 statistic by on average only 0.4% (max = 0.9%). The same is true for the actual likelihood values. This implies that it is relatively easy to distinguish between proteins in different age classes (in particular, very old and very new proteins), but ordering proteins within an age class can be somewhat arbitrary.

### Protein complexes and evolution by duplication

We can test correctness of node anchors identified by DMC and FF using protein annotations. A protein and its duplicate are often involved in similar protein complexes in the cell [37], [46]. We expect then that the node/anchor pairs identified ought to correspond to proteins that are co-complexed. Because it is difficult to model the gain and loss of functional properties of ancient proteins, we only tested this hypothesis among pairs of extant proteins.

Using the MIPS complex catalog [48], which contained annotations for 994 of the proteins in the network, 84% of the testable node/anchor pairs predicted using the DMC model shared an annotation. This is much higher than the baseline frequency: only 55% of edges in the extant network connect nodes that share an annotation. Under the FF model, 68% of node/anchor pairs share a MIPS annotation. So, while the FF model under this validation measure again is performing much better than expected by random chance, it does not perform as well as DMC. The high quality of the DMC-based node/anchor pairs also supports the idea that a good definition of a functional module in a PPI network is one which groups proteins with similar neighbors together (rather than one based strictly on density) [47].

We can also gauge correctness of our node anchors by testing their paralogy. We found that 10% of the extant node anchor pairs predicted by DMC (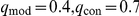) had a pairwise BLAST e-value 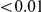. Compared to choosing random pairs of yeast genes (0.002%) and to choosing random pairs connected by an interaction edge (3%), our approach can significantly home-in on likely duplicates. However, many of our predicted duplicates do not correlate with what was predicted by sequence, despite strong evidence from the interaction network. This suggests that the history offered by the network presents a new view on evolution and duplication that can be complementary to the view presented by sequence-based analysis.

**Figure 9:**
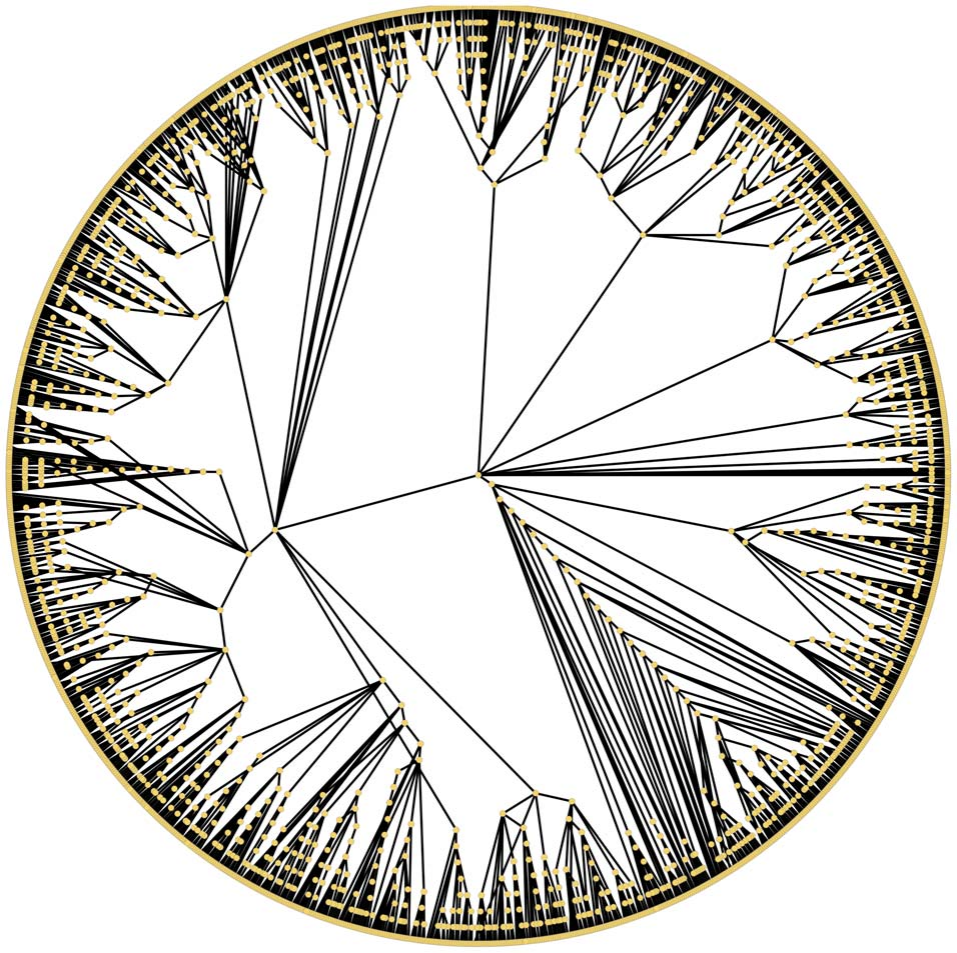
Visualization of the node/anchor phylogeny inferred by reversing the DMC model on the yeast PPI network.

**Figure 10:**
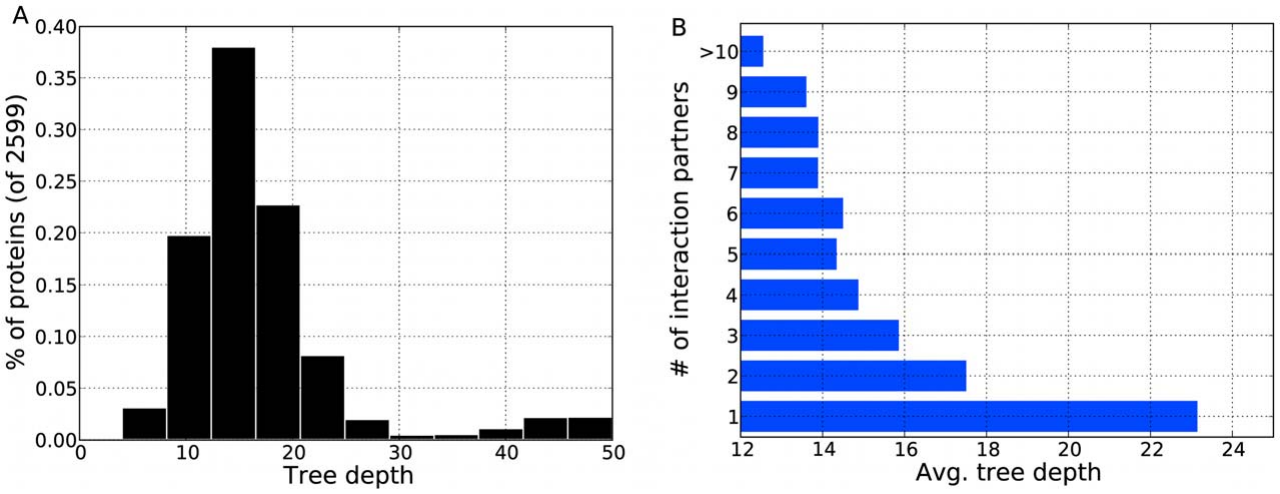
The evolution of protein duplication. (A) The distribution of duplication rates for extant proteins in the PPI network. The 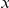 -axis of the histogram is the number of duplications, measured as the distance from the root of the phylogeny to the extant protein. The 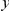 -axis is the percentage of proteins lying in the tree depth bin. (B) The relationship between duplication and number of interaction partners. The 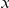 -axis shows the average tree depth for proteins with the given number of interaction partners (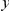 -axis) in 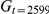. Highly connected proteins tend to duplicate less than proteins with fewer interaction partners.

The phylogeny of node/anchor relationships (Figure 9) can also help characterize how duplication has guided the evolution of the yeast proteome. We estimate the number of times each extant protein was involved in a duplication (that became fixed in the population) by computing the depth of the protein in the inferred node/anchor tree. Figure 10A shows that most proteins are involved in a similar number of duplications (mean = 17, median = 15), with fewer proteins involved in many more or many less. Further, proteins involved in more duplications typically have fewer interaction partners (Figure 10B). Using network histories alone, this correlates with previous sequence-based findings that the evolutionary rate of proteins is inversely proportional to its number of binding partners [33], [34] (though some doubt remains about this fact [49]).

The arrival times of proteins can also tell us how different components of protein complexes might have evolved. For every protein belonging to exactly one MIPS complex, we computed its *coreness*, defined as the percentage of its annotated neighbors that belong to the same complex. A large coreness value indicates that the protein plays a central role in the complex; a small value suggests a peripheral role [50]. Amongst the 763 protein tested, there was a significant correlation between older proteins and larger coreness values (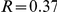, *P* 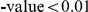), a trend that Kim and Marcotte [32] also independently reported by studying the evolution of protein structure using a different measure of coreness.

The change in clustering coefficient of ancestral networks also hints at how modularity may have evolved. Figure 7B shows that the extant network has a lower clustering coefficient than relatively recent ancestral networks. This could be attributed to the addition of new peripheral components to existing complexes or pathways that evolved to perform functional subtasks [51]. Much older ancestral networks, however, have a smaller clustering coefficient than the extant network, which suggests that some tightly clustered groups were still developing at this time by forming interaction triangles in the network.

### Estimating the arrival of extant and ancestral interactions

Ancestral network reconstruction can also be used to study how interaction edges might have evolved over time. We found that extant edges with recent arrival times (new edges) tended to connect proteins within the same complex versus older edges that tended to connect proteins in different complexes. In particular, 80% of the 100 most recently added extant edges were within-complex edges. This is in stark contrast to the 100 oldest extant edges, of which only 20% were within-complex edges. It is possible that the model confuses purifying selection with recent emergence (i.e. old, conserved events look new); it is also possible that many recent duplications were followed by little divergence, which resulted in the expansion or growth of complexes. The chance that a random extant edge is a within-complex edge is only 53% (std = 2%), which suggests that in either case, there is a significant difference in the topological placement of older and newer extant edges.

Unlike FF and PA, DMC also models edges that were once present in an ancestral version of the network, but that are no longer present in the extant network. These edges are interesting because they hint at structural patterns that were lost over time. We found that many more within-complex edges were modified than we would expect by chance. In particular, 8% of the non-extant, reconstructed edges connected two proteins in the same complex, which is significantly more than the 1.2% found when choosing random non-extant pairs (*P* 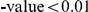). This suggests that modules have re-wired over time.

Studying the relationship between ancient edges and present-day complexes, however, requires some discretion. It is likely that the annotations used today are not reflective of the functional organization of some ancestral networks; new complexes might have emerged, old complexes might have been lost, and interactions that were once within-complex could now be between-complex. Nonetheless, our network reconstruction framework provides a ground from which these questions can be further explored.

### Recovery of past social networks

To contrast the evolution of biological networks with social networks, we applied our algorithms to part of the Last.fm music social network. Edges in this network link users (nodes) that are friends. We snowball-sampled [52] a region of the network by performing a breadth-first crawl starting from a random user ‘rj’. We recorded the date and time of registration for each node visited, which corresponds to its arrival time into the network. The resulting network consisted of the subgraph induced by the first 2957 nodes visited (9659 edges). Because only a subgraph of the complete network was visited, some nodes have neighbors that are outside the induced subgraph. This missing data makes the reconstruction problem even more difficult.

**Figure 11:**
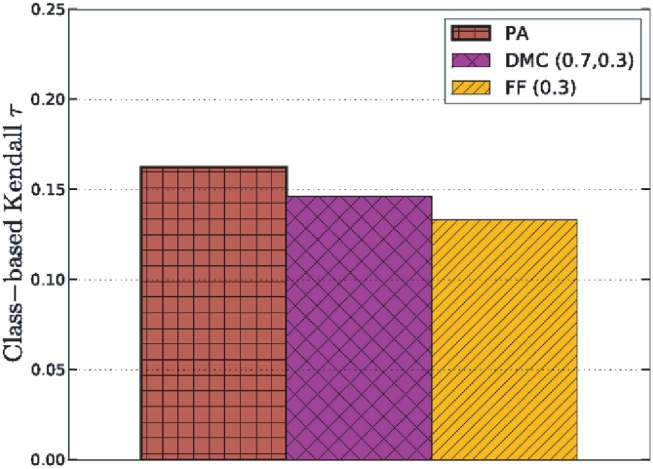
Predicting node arrival times for users in the Last.fm social network. The PA model appears most applicable to reversing the network.

Figure 11 shows the performance of the models (using the best parameters) for the node-arrival measures. The best performing model (preferential attachment) for the Last.fm network was the worse performing model for the PPI network, which confirms the notion that social and biological networks likely grew by different mechanisms [13], [53]. Further, the optimal DMC parameters (0.7,0.3) indicate that new users in social networks form links to a varied set of existing users that might be far apart in the network [10].

An advantage of FF and DMC over PA is that the former return node anchors. To validate these predicted relationships, we make the observation that node/anchor pairs are likely to share similar taste in music. As a null baseline, we computed the percentage of edges in the given network 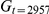 that connect users who share a top-5 favorite artist. The pairs returned by FF are more likely (13.8%) to share a top-5 favorite artist over DMC (10.3%) and the baseline (10.8%). Most users act as anchors to 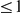 new member, however, there were 9 users who (putatively) each brought 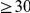 new members into the network. Such popular anchors can be thought of as members who are responsible for the network's organic growth.

## Discussion

We presented a novel framework for uncovering precursor versions of a network given only a growth model by which the network putatively evolved. Our approach works backwards from a given network and is therefore network specific (not model generic) and can retain individual node labels. Unlike heuristic approaches (such as ordering node arrival times based on their static degree in the extant network), our approach reconstructs edges in a principled way, provides a likelihood estimate for ancestral graphs, identifies node anchors, and is driven by a formal mechanism describing network evolution. Further, for most DMC-grown synthetic networks, removal by static degree performs as poorly as PA, as is expected since PA is derived from the assumption that degree is correlated with age [7].

Using the proposed algorithms, we estimated protein ages from the topology of a PPI network alone that matched sequence-based evidence well. Further, we correlated node/anchor pairs with co-complexed proteins and characterized the distribution of duplications on a per-protein basis. We also found that older proteins tend to play a more central role in protein complexes than newer (peripheral) proteins, that recently-arrived edges often formed within existing complexes, and that modules have significantly re-wired over time perhaps by adding peripheral components to their cores. While the true history of the yeast interactome will likely never be exactly recovered, many of these predictions are in agreement with known features of PPI network evolution, which is surprising given the noisy and incomplete status of the available PPI data [54], [55] and the simple network growth models we used. As more complete and accurate networks become available, we can assess how the predictions change by reapplying the proposed algorithms.

We also used the accuracy of history reconstruction as an optimization criterion for choosing model parameters. We determined, via both the DMC and FF models, that duplicated proteins are likely to interact and share many interaction partners. The ability to match the inferred history under a given model to properties of the true history provides an alternative way to validate models that goes beyond comparing only statistics of the final extant network.

A natural extension to this work is to evaluate how the greedy likelihood approach performs on other models [56], such as those that explicitly incorporate an estimate of a node's age [13], [32], those in which nodes can add edges at variable times [12], those that encompass a mixture of several models, or other variations on the PA and DMC processes [13], [39], [40]. Naturally, proteins that emerge via duplication but are eventually lost are also important to model [57]. We found that our algorithms can tolerate some deletion, but additional reversal procedures that explicitly account for deletion are necessary. Automated selection of reverse model parameters and computation of model-based priors to use in the likelihood procedure may also make the reconstructions more accurate and more practical. However, even with the standard models investigated here, our results show that present-day networks are strongly linked to their past, and that this past can be effectively excavated.

## Methods

### Validating node arrival times

Our reconstruction framework gives an ordered list of node arrival times, with the first removed node corresponding to the node that most recently entered. Let 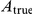 be the true arrival order of the nodes and let 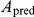 be the computationally predicted sequence. To understand how well our reconstructed arrival times match the true node arrival times, we compute the difference between 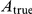 and 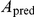 using the popular Kendall's 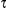 and Spearman's footrule measures [58]:

#### Kendall's tau


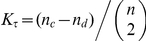
, where 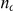 is the number of concordant pairs in 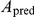, i.e. the number of pairs in 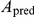 that are in the correct relative order with respect to 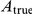; and 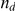 is the number of discordant pairs. 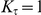 if the two lists are identical, and -1 if they are exactly opposite.

#### Spearman's footrule


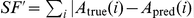
. 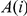 is the node arrival time for node 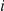. This measure takes into account how far apart the arrival times are for each node in the two lists. 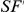 has a maximum value of 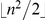. We use a normalized value of 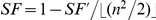, so that 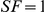 if the two lists are identical, and 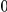 if they are opposite of each other.

In both cases, the higher the value the better. The expected 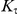 and 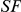 similarity between 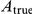 and a random ordering of the nodes is 0.00 and 0.33, respectively.

### Validating node anchors

**Figure 12:**
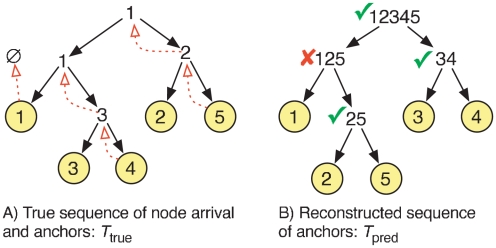
Computing the similarity of node/anchor pairs in the true versus the reconstructed histories.

When a node enters the network under the DMC and FF models, it chooses an existing node from which it copies links. We call this node its *anchor*. To assess our ability to identify node/anchor relationships, we encode the true node/anchor relationships in a binary tree. We can think of a node's arrival as causing its chosen anchor node to divide in two, producing a new node and a new copy of the old node. Figure 12A shows a binary tree describing such a bifurcation process, with node anchors indicated by dotted arrows. In this example, node 1 initially exists alone in the network, and therefore has no anchor. Reading from top down, node 2 enters and chooses node 1 as its anchor. This spawns a new node 1, which is conceptually different from its parent because the new node could have gained or lost edges due to the arrival of node 2. Node 3 enters and chooses the new node 1 as its anchor. Finally, nodes 4 and 5 anchor from nodes 3 and 2, respectively.

Figure 12B shows an example sequence of merges predicted by our reconstruction algorithms. Internal nodes in the tree are labeled with the concatenation of the labels of its two children indicating an inferred node/anchor relationship between the children.

Let 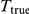 be the anchor tree derived from the true growth process (Figure 12A) and let 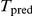 be the reconstructed anchor tree (Figure 12B). To assess the quality of the reconstruction, we compute the percentage of subtrees in 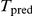 found in 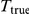. This measure (called **Anchor**) is closely related to the Robinson-Foulds distance metric used to compare phylogenetic trees [31]. In the example of Figure 12, the similarity between the trees is 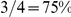.

This validation measure is advantageous because it evaluates if the relationship between larger groups of nodes was correctly determined. In addition, it does not unduly penalize the mis-ordering of arrival times for nodes that are far apart in the network. It also does not depend on which node of the merged pair 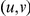 was deleted from the graph in the DMC model, because both choices lead to the same subtree in 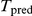. On the other hand, the measure is in some ways stricter than counting correct node/anchor pairs. For example, in Figure 12 it would be incorrect to merge 1 and 2 in the first backward step because the extant nodes 1 and 2 are not the same as the past nodes 1 and 2.

### Availability

Our code and data are available online at http://www.cbcb.umd.edu/NetArch.
